# Treatment Efficacy for Androgenetic Alopecia (Hair Loss): Combination Therapy of Clinical Low‐Level Laser (Light) Therapy and Proprietary Hair Growth Serum: A Pilot Study

**DOI:** 10.1111/jocd.71195

**Published:** 2026-10-01

**Authors:** Garrett Ni, Megan Saltsgaver, Gabrielle Cahill, Robert Kang

**Affiliations:** ^1^ Division of Facial Plastic & Reconstructive Surgery, Department of Otolaryngology – Head and Neck Surgery University of California Los Angeles Los Angeles California USA; ^2^ University of Illinois College of Medicine Rockford Illinois USA

**Keywords:** androgenetic alopecia, growth serum, hair health, low‐level laser therapy

## Abstract

**Introduction:**

Androgenetic alopecia (AGA) is a common cause of hair loss with limited non‐surgical treatment options. Low‐level laser therapy (LLLT) and topical serums are emerging as promising therapies. This study evaluates the combined efficacy of Sunetics Clinical LLLT and a proprietary hair growth serum in promoting hair regrowth.

**Methods:**

A prospective study was conducted on 8 patients (5 males, 3 females; mean age 56.4) with AGA. Each underwent 24 Clinical LLLT treatments over 3 months, along with 3 monthly applications of the proprietary hair growth serum delivered using IonFuse needleless infusion. Objective hair metrics were analyzed using the Canfield HairMetrix system. Parameters measured included hair density, terminal‐to‐vellus hair ratio, hair width, and follicular unit characteristics.

**Results:**

Significant improvements were observed after 3 months. Hair count/cm^2^ increased by 25.45%, hairs per follicular unit by 42.27%, terminal‐to‐vellus ratio by 24.51%, and average hair width by 3.53%. Follicular unit density improved by 14.01%. Subjective improvements were modest, though all participants found the treatment tolerable and would recommend it.

**Conclusion:**

The combination of Clinical LLLT and the proprietary hair growth serum appears effective and well‐tolerated for AGA treatment. While early results are encouraging, larger, controlled studies are necessary to validate findings and assess long‐term efficacy.

## 1 Introduction

Low‐level laser (light) therapy (LLLT) is an innovative treatment gaining popularity for its potential benefits in promoting hair growth. By utilizing specific wavelengths of light, LLLT stimulates cellular activity and enhances blood flow to the scalp, which can help rejuvenate hair follicles and encourage new hair growth [1, 2, 3, 4, 5]. This non‐invasive therapy is not only safe, but also convenient. Making it an appealing option for those experiencing hair thinning or loss. As research continues to uncover the mechanisms behind its effectiveness, many individuals are turning to LTTT as a promising solution in their hair restoration journey. Whether used alone or in combination with other treatments, LLLT offers a hopeful avenue for enhancing hair health and vitality.

In addition to LLLT, traditional topical therapy, such as Minoxidil, has gained popularity for its remarkable ability to stimulate hair follicles and promote new hair growth. Along with Minoxidil, a variety of serums, oils, and specialized shampoos are available, each offering unique benefits.

U.SK Skin Savers Hair Growth (San Diego, CA) is an alternative topical treatment targeting androgenetic alopecia. Combining advanced scientific formulations with nourishing ingredients, these products target the scalp to revitalize hair follicles and create an optimal environment for growth. In our experience, we have seen improvement in hair loss when patients use the U.SK Skin Savers Hair Growth serum.

This pilot study examines the effectiveness of LTTT combined with the U.SK Hair Growth serum at promoting hair growth.

## 2 Methods

The Cottage Health Institutional Review Board approved this prospective pilot case series (approval number: 24‐57ix). Written informed consent was obtained from all participants prior to enrollment.

Patients were eligible for inclusion if they were 18 years of age or older and had clinically diagnosed androgenetic alopecia confirmed by the senior author. Patients were required to be willing to complete the 3‐month treatment protocol and follow‐up visits. Exclusion criteria included pregnancy, incarceration, inability to provide informed consent, active scalp infection or inflammatory scalp disease, and any medical condition that, in the investigator's judgment, would interfere with study participation or outcome assessment. Patients receiving other medical treatments for androgenetic alopecia were required to discontinue these therapies prior to enrollment. Patients with a prior history of follicular unit transplantation were eligible provided no additional hair restoration procedures were performed during the study period.

Prior to initiating treatment, standardized baseline photographs of the scalp were obtained using the Canfield D200 HairMetrix system (Canfield Scientific, Fairfield, NJ). The Canfield HairMetrix system is a non‐invasive, AI‐powered digital imaging and analysis platform designed to evaluate hair and scalp characteristics with high precision. Utilizing cross‐polarized and non‐polarized photography, HairMetrix captures high‐resolution images that are analyzed to quantify hair density, hair diameter, follicular unit composition, and miniaturization.

Images were taken from areas of scalp with least hair density upon initial inspection. To ensure consistency and reproducibility in image acquisition, naturally occurring scalp landmarks, such as small skin lesions or benign pigmented lesions, were used to standardize the imaging location. These anatomical landmarks allowed consistent imaging of the same scalp region at baseline and follow‐up. All images were acquired by the first author using the same imaging system and standardized acquisition protocol throughout the study.

Patients underwent treatment with the Sunetics Clinical Hair Growth Laser (Sunetics, Southlake, TX) combined with hair growth serum therapy for 3 months. Treatments were performed twice weekly. Each session consisted of 30 min of low‐level laser therapy (LLLT), for a total of 24 treatments over the study period.

The Sunetics Clinical Hair Growth Laser is an FDA‐cleared, Class II, in‐office LLLT system indicated for androgenetic alopecia. The device utilizes a multi‐diode array in a fixed overhead configuration designed to provide uniform, full‐scalp coverage without repositioning. An optical beam dispersion design distributes low‐level laser energy evenly across the treatment area, minimizing variability in irradiance. The system emits coherent red light at 650 nm (±5 nm) through AlGaInP semiconductor diodes, each operating at approximately 5 mW in continuous‐wave mode.

Additionally, 5 mL of the U.SK Hair Growth Serum was applied to the scalp once monthly for a total of three applications during the study. The U.SK Under Skin Hair Saver serum contains ingredients including Octapeptide‐2, Copper Tripeptide‐1, and VEGF. The serum was delivered using the IonFuse needleless infusion system, which employs microcurrent‐assisted transdermal delivery to enhance penetration into the scalp.

The primary study endpoint was completion of the 24th LLLT treatment at 3 months. Repeat HairMetrix imaging was performed at this visit. Primary outcome measures included hair count/cm^2^, terminal‐to‐vellus hair ratio, average hairs per follicular unit, average hair width (μm), follicular units/cm^2^, and average interfollicular distance (mm).

Baseline demographic and clinical information, including age, sex, comorbidities, medications, and prior hair restoration procedures, were obtained by chart review. Adverse events were assessed throughout treatment and at follow‐up visits, including scalp irritation, discomfort, excessive shedding, and other treatment‐related reactions.

## 3 Results

A total of 8 patients completed the full 3 months of treatment. The mean age was 56.4 (range 43–63); there were 5 males and 3 females. The average Norwood scale was 2.38 and the cohort had an average Fitzpatrick scale of 2.5. In terms of comorbidity and current pertinent treatments to hair health, 2 of the 8 patients had a history of follicular unit transplantation. No patients were actively on medical treatments for androgenetic alopecia at the time of the study. There were no adverse events during the treatments or at the 3 month follow up visit.

The mean before and after results are as follows: hair counts per cm^2^ were 110 and 138 (25.45% increase), mean terminal to vellus ratio 19.59 and 24.33 (24.51% increase), mean hair per follicular unit were 1.21 and 1.72 (42.27% increase), mean hair width 65.08 and 67.38 μm (3.53% increase), follicular units per cm^2^ 91 and 103 (14.01% increase), and mean interfollicular distance 1.14 and 1.135 (0.33% decrease) (Table 1, Figures 1 and 2).

**TABLE 1:** Pre‐ and post‐treatment Canfield HairMetrix results.

Patient	Hair count/cm^2^		Sum hair width/cm^2^		T:V ratio (*n*:1)		Hair/FU		Avg hair width		FU/cm^2^		Interfollicular distance	
	Before	After	Before	After	Before	After	Before	After	Before	After	Before	After	Before	After
1	143	204	7977	10 829	12.1	12.6	1.2	1.2	55.8	53	105	151	1.05	0.91
2	133	143	10 374	11 668	61	65	1.3	4.4	78	81.6	93	110	1.14	1.05
3	162	189	8942	11 472	6.81	14.2	1.3	1.5	55.2	60.7	101	129	1.08	1.09
4	108	118	7052	7965	11.5	12.4	1.3	1.6	65.3	67.5	89	91	1.15	1.19
5	89	90	6353	6669	4.4	6.6	1.1	1.3	72.2	74.1	64	68	1.20	1.24
6	83	97	4996	6266	18.7	37	1.2	1.3	60.2	64.6	79	65	1.34	1.42
7	122	132	9211	10 085	34	38.3	1.1	1.2	75.5	76.4	105	115	1.03	1.04
8	129	131	7546	8017	8.2	8.5	1.2	1.3	58.5	61.2	92	101	1.12	1.14
Mean	110.00	138.00	7806.38	9121.38	19.59	24.33	1.21	1.73	65.09	67.39	91.00	103.75	1.14	1.14
% Δ	+25.45 (p=0.342)		+16.85 (p=0.198)		+24.51(p=0.641)		+42.27 (p=0.206)		+3.53 (p=0.627)		+14.01 (p=0.285)		−0.33 (p=0.954)	

**FIGURE 1:**
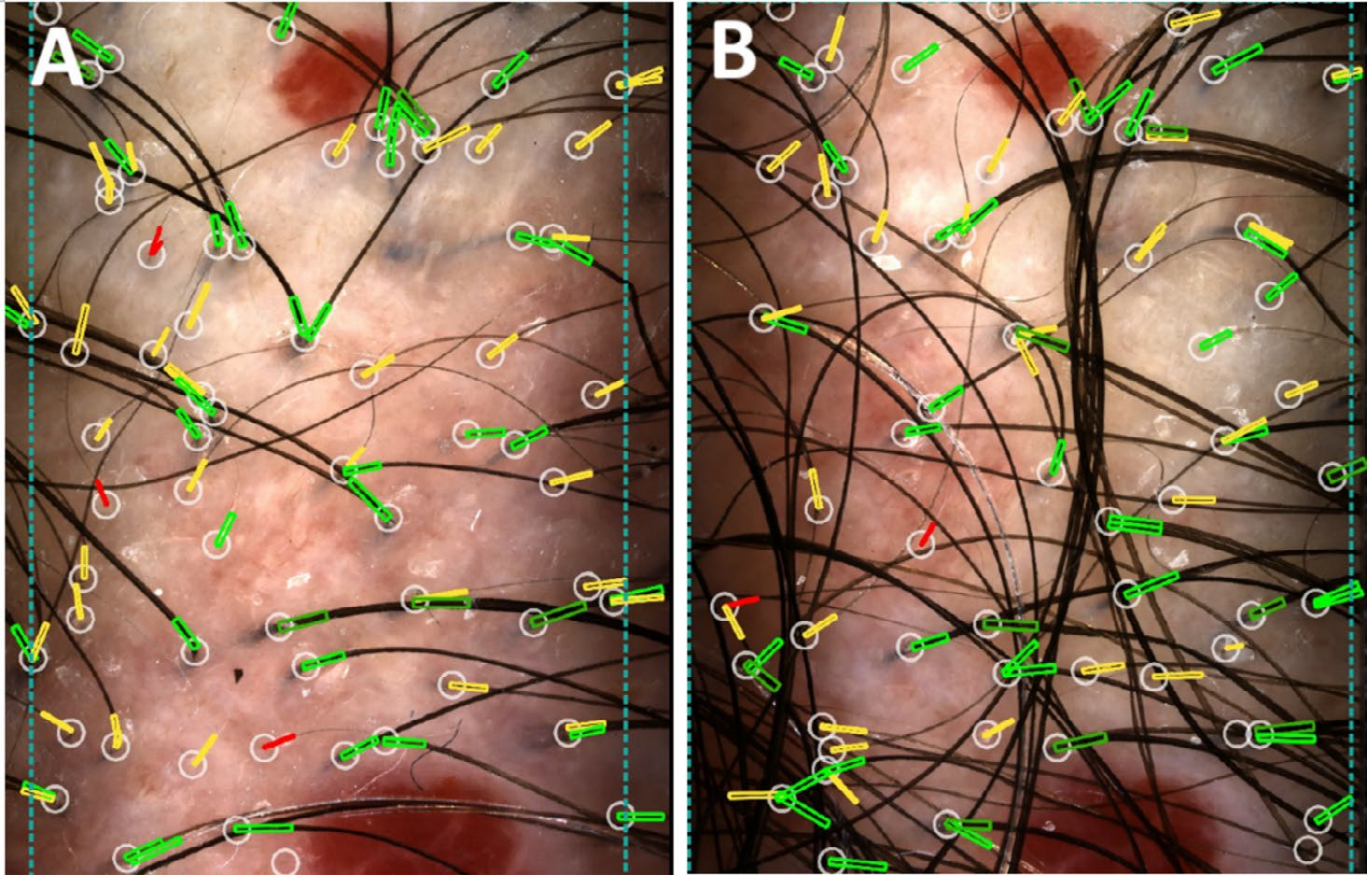
Canfield hair analysis of a 63 years old patient before (A) and after 3 months of treatment (B).

**FIGURE 2:**
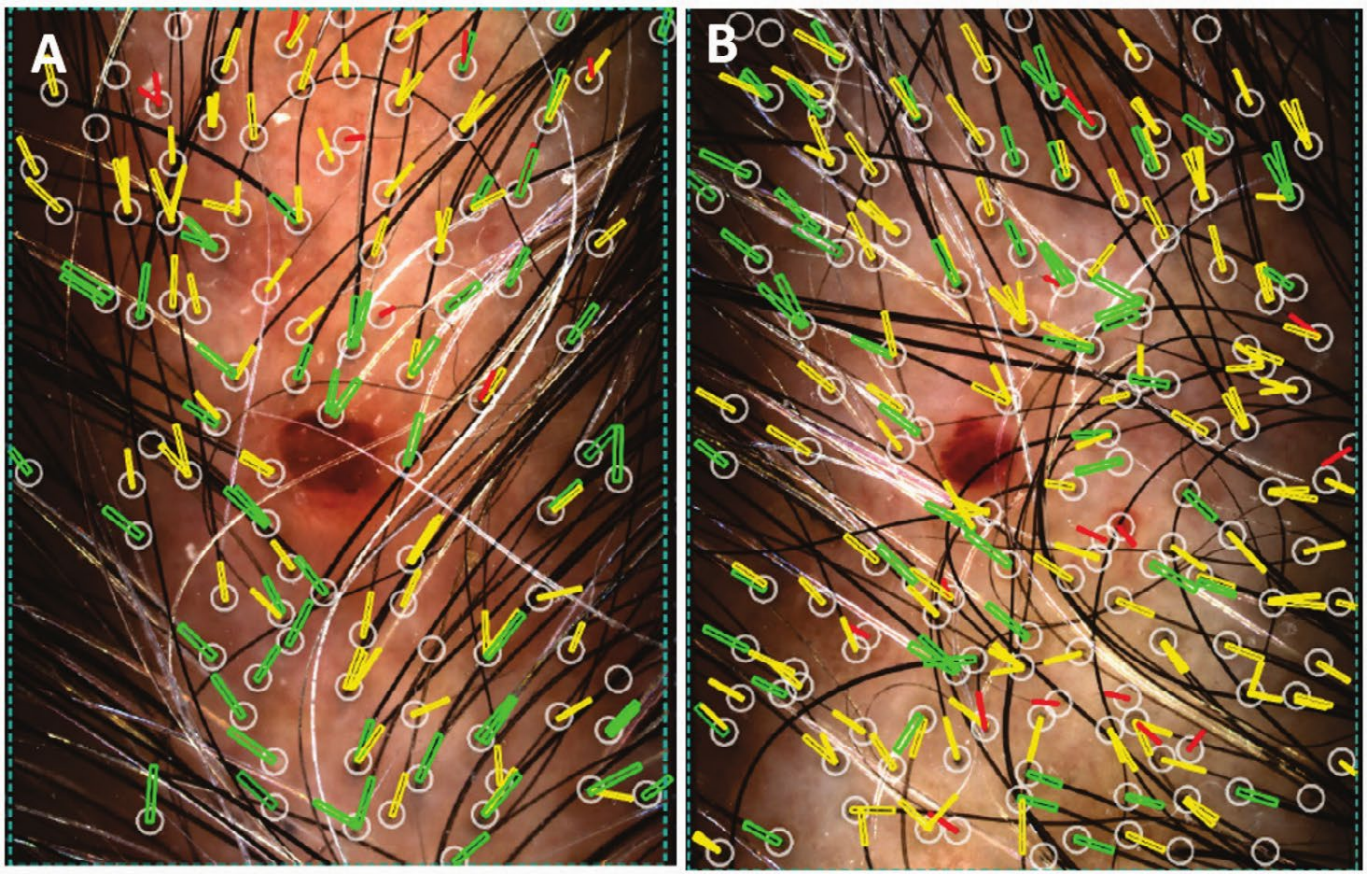
Canfield hair analysis of a 56 years old patient before (A) and after 3 months of treatment (B).

## 4 Discussion

The findings of this prospective pilot case series suggest that combination treatment with low‐level laser therapy (LLLT) and the U.SK Hair Growth Serum may improve objective hair parameters in patients with androgenetic alopecia. Over the 3‐month treatment period, improvements were observed in several objective hair metrics, including total hair count, terminal‐to‐vellus ratio, hair width, and follicular unit density.

Most notably, the average number of hairs per follicular unit increased by 42.27%, and hair count per cm^2^ increased by 25.45%, both suggesting improved follicular productivity over the treatment period. While the mean hair width increased modestly by 3.53%, this finding may reflect early thickening of miniaturized hairs.

These findings are generally consistent with the contemporary literature evaluating LLLT and photobiomodulation for androgenetic alopecia. A recent systematic review concluded that LLLT is associated with improvements in hair density across randomized clinical trials and that treatment parameters, including energy fluence, duration of exposure, and light delivery characteristics, may influence clinical outcomes. Additionally, another study demonstrated that a 24‐week randomized trial demonstrated significant improvements in hair density with an excellent safety profile. Although direct comparisons are limited by differences in treatment protocols, study design, and follow‐up duration, the objective improvements observed in our cohort over 12 weeks are consistent with the growing body of evidence supporting light‐based therapies as a safe and potentially effective treatment modality for androgenetic alopecia [6, 7].

Topical minoxidil remains one of the standard non‐surgical treatments for androgenetic alopecia; however, its effectiveness depends on long‐term adherence, which may limit patient compliance. Finasteride is another FDA‐approved treatment option but may be associated with adverse effects that limit its use in some patients [8]. Previous studies evaluating the combination of minoxidil and LLLT have not consistently demonstrated additional benefit over short treatment periods [9, 10]. While there is limited peer‐reviewed evidence specifically evaluating the U.SK Hair Growth Serum, its formulation including peptides and growth factors resembles other topical cosmeceutical products investigated for hair restoration. For example, Lee et al. demonstrated improvements in hair density following topical growth factor therapy over a 3‐month treatment period [11]. Although objective improvements were observed following combination therapy in our study, the concurrent administration of both LLLT and the serum precludes determination of the individual contribution of either intervention. Consequently, any additive or synergistic effect remains speculative and should be evaluated in future controlled studies.

In addition to LLLT and topical therapies, regenerative approaches such as platelet‐rich plasma (PRP) and hair follicle stem cell (HFSC)‐based therapies have demonstrated encouraging improvements in hair density and thickness in patients with androgenetic alopecia. However, substantial variability exists among treatment protocols, outcome measures, and follow‐up duration, making direct comparisons across studies difficult. Future comparative studies evaluating combination LLLT, topical biologic therapies, PRP, and regenerative strategies may help define the relative efficacy of these treatment modalities.

In contrast to the objective improvements observed, patients' subjective assessments were more conservative. Most participants were neutral regarding visible hair growth, perceived slowing of hair loss, and overall improvement in hair appearance. However, Leavitt et al. demonstrated significant patient‐perceived improvement following 26 weeks of LLLT treatment [12]. Despite the modest subjective responses in our cohort, all participants found the treatment regimen well tolerated and indicated that they would recommend it to others. These findings underscore the importance of managing patient expectations while also supporting the favorable safety profile and patient acceptability of the treatment regimen. This is consistent with previous studies demonstrating that LLLT is generally well tolerated with minimal reported adverse events [13, 14].

This pilot study has several limitations. The sample size was small, follow‐up was limited to 3 months, and no untreated or active comparator group was included. In addition, because all participants received both LLLT and the U.SK Hair Growth Serum, the independent contribution of each treatment cannot be determined. Hair imaging and analysis were also performed by the same unblinded investigator, introducing the potential for measurement bias despite the use of standardized imaging protocols and objective HairMetrix measurements. Accordingly, these findings should be considered preliminary. Future studies with larger cohorts, longer follow‐up, and randomized controlled designs comparing individual and combination treatment modalities are needed to confirm these observations, determine the relative contribution of each intervention, and evaluate the durability of treatment response.

## 5 Conclusion

The combined use of LLLT and U.SK Hair Growth serum appears to offer a well‐tolerated approach for individuals experiencing hair thinning or androgenetic alopecia. While early results are encouraging, further investigation is warranted to fully understand the synergistic mechanisms and long‐term efficacy of this dual‐modality treatment.

## Author Contributions

**Megan Saltsgaver:** drafting of manuscript, revision of manuscript. **Gabrielle Cahill:** revision of manuscript. **Garrett Ni:** data curation and analysis, drafting of protocol and manuscript, revision of protocol and manuscript, project supervision. **Robert Kang:** data curation and analysis, drafting of protocol and manuscript, revision of protocol and manuscript, project supervision.

## Funding

The authors have nothing to report.

## Conflicts of Interest

The authors declare no conflicts of interest.

## Data Availability

The data that support the findings of this study are available on request from the corresponding author. The data are not publicly available due to privacy or ethical restrictions.
